# It Runs in the Family: Testing for Longitudinal Family Flynn Effects

**DOI:** 10.3390/jintelligence11030050

**Published:** 2023-03-06

**Authors:** Linda Wänström, Patrick O’Keefe, Sean A. P. Clouston, Frank D. Mann, Graciela Muniz-Terrera, Stacey Voll, Yun Zhang, Scott M. Hofer, Joseph L. Rodgers

**Affiliations:** 1 Department of Computer and Information Science, Linköping University, 581 83 Linköping, Sweden; 2 Department of Neurology, Oregon Health and Science University, Portland, OR 97239, USA; 3 Program in Public Health and Department of Family, Population, and Preventive Medicine, Stony Brook University, Stony Brook, NY 11794, USA; 4 Ohio University Heritage College of Osteopathic Medicine (OUHCOM), Dublin, OH 43016, USA; 5 Edinburgh Dementia Prevention, University of Edinburgh, Edinburgh EH4 2XU, UK; 6 Institute of Aging and Lifelong Health, University of Victoria, Victoria, BC V8N 1V8, Canada; 7 Department of Psychology and Human Development, Vanderbilt University, Nashville, TN 37232, USA

**Keywords:** family Flynn effect, IQ, intelligence, NLSY, multilevel growth curve models

## Abstract

The Flynn effect refers to increases over time in measured (particularly fluid) intelligence of approximately 3 IQ points per decade. We define the Flynn effect at the family level, using longitudinal data and two new family-level cohort definitions. Multilevel growth curve analyses of the National Longitudinal Survey of Youth 1979 data showed that children in families with later-born mothers had higher average PIAT math scores, and lower average reading comprehension scores and growth, in young and middle childhood. Children in families where the first child was born later had higher average PIAT math, reading recognition, and reading comprehension scores, as well as larger developmental growth. The latter family-level Flynn effects were of higher magnitudes than the usual individual-level Flynn effect found in previous studies. Our results, showing family level-intercept and slope Flynn effects for both maternal birthyear and first child birthyear, have implications for research aiming to explain the Flynn effect.

## 1. Introduction

Almost 40 years ago, James Flynn (1984) revived interest in studying the secular increase in intelligence that was occurring in the U.S. (see Lynn 2013, for a history). Following this, thousands of research studies have documented and attempted to explain increasing IQ scores across countries, ages, and different IQ measures. Comprehensive meta-analyses are presented in Pietschnig and Voracek (2015) and Trahan et al. (2014). In the first 20 years after Flynn’s first study, a number of Flynn effect discoveries were published, as well as a number of explanatory models that attempted (without full success) to provide explanations for the empirical patterns. A relatively recent Flynn effect discovery was the finding that the pace may be slowing down—or even reversing—in some parts of the world (see Teasdale and Owen 2005). However, recently, the pace of discovery has stagnated. 

This paper documents new Flynn effect patterns. Few past studies have questioned the “location” of the Flynn effect in relation to the family. Rather, past work has strongly focused on individual-level patterns to document the Flynn effect. In addition, few past studies have investigated the Flynn effect longitudinally, examining the presence of Flynn effects on not only differences in intelligence levels between cohorts, but also differences in intelligence development between cohorts. In the current study, we evaluate whether the Flynn effect exists at the family level, and whether it exists in both levels and in growth. A Flynn effect in levels means that later-born cohorts have higher IQ levels than earlier-born cohorts. A Flynn effect in growth means that later-born cohorts are increasing faster in IQ than earlier-born cohorts. In the remainder of the introduction, we review past Flynn effect studies, and present our empirical analysis. 

The Flynn effect refers to secular increases in measured intelligence—particularly fluid intelligence, the type of intelligence associated with problem-solving—that have been documented globally, and have occurred for more than a century. The average increase during the 20th century was approximately 3 IQ points per decade. Flynn (1984, 1987) noted that individuals belonging to later generations tended to score higher on the same IQ tests than those who took the tests years earlier, and individuals taking several tests, normed at different times, tended to score higher on tests that were normed earlier. In the past few decades, and in some locations, the pace of the Flynn effect has appeared to be slowing, or even reversing (Dutton and Lynn 2013; Sundet et al. 2004; Teasdale and Owen 2000; however, different patterns were found by Gonthier et al. 2021; O’Keefe and Rodgers 2020). The research community has reached no broad consensus on the causes of the Flynn effect, or for this potential slowing trend (Rindermann et al. 2016). Over a dozen face-valid, empirically defensible explanations exist; however, each explanation has empirical/theoretical weaknesses. Pietschnig and Voracek’s (2015) meta-analysis suggested that Dickens and Flynn’s (2001) social multiplier (niche-picking) theory, and the life history perspective (e.g., Woodley et al. 2013), had the most support. Other popular mechanisms have included nutrition (e.g., Lynn 2009), education (e.g., Baker et al. 2015; Williams 1998), technology (e.g., Neisser 1997), testing effects (e.g., Fuggle et al. 1992), health and health care services (e.g., Steen 2009), and heterosis (e.g., Mingroni 2007). A number of broad summaries of these theories have been published (e.g., Ang et al. 2010; Pietschnig and Voracek 2015). 

Rodgers (1998) suggested that research on the Flynn effect had moved forward more aggressively than the empirical understanding of the effect could justify. Almost two decades later, Rodgers (2015) was more sanguine about the knowledge base, because of a closer match between the empirical evidence and the theories that had been proposed. Nevertheless, he still raised important methodological and empirical issues, such as empirically separating within- and between-family variance, and using growth curve models, when studying the Flynn effect. The current paper addresses these concerns by studying the Flynn effect longitudinally and at the family level.

Most previous studies have evaluated the Flynn effect at the individual level, ignoring family units, although some studies have distinguished between within- and between-family variance. Sundet (2014) and Bratsberg and Rogeberg (2018) found Flynn effect patterns inside families, across siblings, in Scandinavian data. Rodgers (2014) used data from Belmont and Marolla (1973) to show that the Flynn effect could be the cause of empirically observed, cross-sectional, within-family birth order effects, when measured in cross-sectional studies. O’Keefe and Rodgers (2017) used U.S. data from the National Longitudinal Survey of Youth (NLSY79), based on a household probability sample of U.S. adolescents in 1979, and found that the Flynn effect was most strongly linked to between-family patterns. When the child cohort variable in the NLSY-Children (NLSYC) data (the children born to NLSY79 females) was separated using multilevel models into between-family, within-family, and within-individual components, most of the effect was located in between-family patterns. Finding Flynn effects at the family level, i.e., with family as the unit of measurement, may have implications for theories of the causes of the Flynn effect. In the present study, we investigate the presence of a family-level Flynn effect, both on levels and slopes of growth curves, using children and mothers from the NLSY79 data. Importantly, in the current study, new measures of cohort at the family level are defined.

When investigating the Flynn effect at the individual level, previous studies have used the individual’s birthyear (or birth decade, etc.) as the cohort measure. When family is the unit of interest, defining a cohort is not quite as straightforward. O’Keefe and Rodgers (2017) studied components of child and maternal birthyears and ages, including the age of the mother at the birth of her first child, as control variables (though not to define cohorts). In the current study, we use two definitions, both relevant to the idea of measuring the family cohort: the birthyear of the mother, and the birthyear of the firstborn child (note that the difference between these two measures is maternal age at first birth, a common and often-used variable in the psychological and demographic literature; e.g., Rodgers et al. 2008; Neiss et al. 2002). We use these family-level cohort measures because they are defined at the family level, and reflect between-family variance (which was identified as the primary location of the Flynn effect in the NLSY data by O’Keefe and Rodgers 2017). They are substantively interesting in relation to a family-level Flynn effect. The former variable defines the cohort of (one of) the parents. If it is parental characteristics that determine the family outcomes, this is a plausible cohort variable to capture such effects. The second variable defines the beginning of parenting for a family. If it is the timing of parents’ entry into parenthood that determines family outcomes, this cohort variable is more appropriate. Our design differs from O’Keefe and Rodgers’ (2017) in our explicit definitions of the two family cohort variables, and in our longitudinal focus, by studying the presence of family Flynn effects (using both family cohort variables) in both the levels and slopes of the family growth trajectories. Our analyses also incorporate more outcome measures (the Peabody Individual Achievement Test (PIAT) math, reading recognition, and reading comprehension subtests). Our approach is different from most earlier Flynn effect studies using the NLSY, where child-level birth cohorts defined the Flynn effect, rather than family birth cohorts. 

Because James Flynn argued in his early papers (e.g., Flynn 1984, 1987) for a cohort-based interpretation of the observed changes in intelligence, most theories have (implicitly or explicitly) searched for causes of the effect that are cohort-based. Whether the causes of the Flynn effect are predominantly cohort, period, or aging phenomena (or some combination of these) has seldom been addressed however (see critique relevant to this issue in Clouston et al. 2021; Rodgers 1998). The methodology for separating these three (confounded) processes with regard to the Flynn effect, or other processes in general, is limited in key ways (Bell and Jones 2013; Fienberg 2013; Fosse and Winship 2019a; O’Keefe et al. 2022), although models are estimable with constraints (Fosse and Winship 2019b; Keyes et al. 2010). Most past Flynn effect studies have used cross-sectional designs, in which cohort and aging effects are especially difficult to disentangle. Giangrande et al. (2022) noted several problems in cross-sectional studies of the Flynn effect. As an example, Dickinson and Hiscock (2010) used the WAIS in a U.S. sample to compare 20- and 70-year-olds in terms of verbal and performance IQ. The younger sample out-performed the older sample, providing support for an aging interpretation. However, in this (and other) samples, aging and cohort effects are confounded by the cross-sectional nature of the dataset. When they adjusted for a Flynn effect at the cohort level, they concluded that around 85% of the apparent aging decline between ages 20 and 70 was attributable to the cohort change, whereas only 15% was attributable to aging, suggesting that the Flynn effect can “stand in” for an aging effect. 

The Flynn effect may show different magnitudes depending on the ages of the individuals studied (e.g., Pietschnig and Voracek 2015). Rodgers and Wänström (2007) noted tendencies toward larger Flynn effects for children of older ages when they compared PIAT Math scores among different birth cohorts of 5-year-olds, different birth cohorts of 6-year-olds, and so on up to different birth cohorts of 13-year-olds. Kanaya et al. (2005), on the other hand, found smaller effects for older children when they studied WISC scores of students who tested for admittance to special education. The latter corresponds to the findings of Shakeel and Peterson (2022), who found larger effects for younger children when analyzing the results of math and reading tests. In contrast, Salthouse (2015), studying adults, did not find any differential Flynn effects at different ages. In order to separate cohort and aging effects, the Flynn effect should be studied longitudinally, which has been done in some studies. For example, Skirbekk et al. (2013) used data from the English Longitudinal Study of Ageing survey and found greater cognitive improvement among later cohorts, whereas Karlsson et al. (2015) found faster declines with aging for later Swedish cohorts. Giangrande et al. (2022) estimated latent growth factors (intercept and slope), and found Flynn effects in the levels (intercepts) and in the growth (slopes) for both fluid and crystallized measures, suggesting that the later cohorts scored higher on average, but also had steeper growth (for ages 7–15). They stressed the importance of studying the Flynn effect both between and within individuals in order not to miss effects on cognitive development, and noted that multilevel models are suitable for this purpose. In order to study longitudinal Flynn effects, we will use raw scores from PIAT measures, using multilevel growth curve models. This will enable us to study Flynn effects in both the score levels and in the slopes of trajectories, which enables us to detect differential growth effects for different family cohorts. Previous studies on Flynn effects in the NLSY (Ang et al. 2010; O’Keefe and Rodgers 2017) have used age-normed scores and have thus not been able to detect effects on development (Rodgers and Wänström 2007, analyzed both normed and raw scores, but did not analyze growth).

To summarize, the past century has shown increases in measured intelligence across cohorts (Flynn effects); however, most previous research has studied the Flynn effect at the individual level, using cross-sectional data. In the present study we investigate the Flynn effect at the family level (using a different design and different definitions of cohort than those in O’Keefe and Rodgers 2017). Our design is longitudinal, using children belonging to the same family, examining both differences in levels and slopes of family trajectories. We use two definitions of family cohorts: maternal birthyear and first child birthyear. The significance of this research is to further expand the focus of the Flynn effect from the individual to the family, to develop new ways to operationalize what a cohort effect is in relation to the family, and to investigate the Flynn effect in developmental slopes in addition to levels. Each of these goals pushes research on the Flynn effect in new directions, methodologically and empirically.

## 2. Materials and Methods

### 2.1. Data

The National Longitudinal Survey of Youth 1979 (NLSY79) (Bureau of Labor Statistics and U.S. Department of Labor 2019a) is an ongoing longitudinal survey based on a household probability sample of 12,686 adolescents and young adults in the US between the ages 14 and 21 at the end of 1978. The NLSY-Children (NLSYC) (Bureau of Labor Statistics et al. 2019b) are the biological children of the mothers in the NLSY79 and they have been surveyed every other year since 1986. The children (51% males, 49% females; 53% non-Black/non-Hispanic, 28% Black, 19% Hispanic or Latin) were cognitively assessed using the Peabody Individual Achievement Test (PIAT) mathematics, reading recognition, and reading comprehension subtests every other year starting at age 5 until they reached age 15, between 1986 and 2014. Thus, the children have repeated measurements on these subtests (assessments at ages 5, 7, 9, 11 and 13, or at ages 6, 8, 10, 12 and 14). The mean assessment age was 9.75. The NLSYC respondents were born between 1970 and 2009, with a majority (50%) born between 1982 and 1991. 

Because our aim is to study the family as a unit, longitudinally, using the NLSY79 and NLSYC datasets serves our purposes well, because all children (of the appropriate assessment ages) of the mothers of the original NLSY79 sample were assessed up to five times for a long period of time (1986 until 2014), making most families complete with regards to child-rearing. Using child siblings instead of adult siblings is also preferable, with regard to our aims, because children share their environments with their siblings to a much greater extent than do adult siblings, making the definition of a family more appropriate. Approximately 25.7% of the families had one cognitively assessed child, 39.9% had two, 22.0% had three, 8.4% had four, 2.7% had five, and the rest had six to ten assessed children. Because the mothers of the NLSYC children were born between 1957 and 1964, our maternal birthyear cohort variable ranges from 1957 to 1964. The first NLSYC firstborn child was born in 1970 and the last firstborn child was born in 2007, so our first child birthyear cohort variable ranges from 1970 to 2007.

We used the PIAT measures math, reading recognition, and reading comprehension for our analyses in this study. Previous studies have found child Flynn effects using an individual-level cohort definition in the NLSYC for PIAT math (Ang et al. 2010; Rodgers and Wänström 2007) as well as between family effects for PIAT math (O’Keefe and Rodgers 2017). The child Flynn effects in PIAT reading recognition and reading comprehension were of smaller magnitudes, and typically reduced towards zero when controlling for maternal IQ (Rodgers and Wänström 2007). We include all three PIAT measures in the current study because our family analyses use family-level cohort definitions, a different design, and different scalings of the response variables as compared to the previous studies. There was a total of 11,530 children in the NLSYC dataset. Out of these, 9233 were assessed with the PIAT math test, resulting in a total of 4055 mothers with 9233 children and 34,498 measurements used in our PIAT math analyses. The corresponding numbers for PIAT reading recognition were 4051 mothers with 9220 children and 34,358 measurements, and for PIAT reading comprehension, 4046 mothers with 9199 children and 33,655 measurements.

All the children’s PIAT math scores come from the same instrument of 84 items, increasing in difficulty, and used across all ages. The starting point on the instrument increases for each age; if a test-taker misses the first several items, they move back to the starting point for the previous age. Once a respondent correctly answers five items in a row, the first item is established as the “basal”. Respondents continue from the basal as long as they get a subset of items correct, and finish when they miss five out of seven items. Their PIAT math score is the item number of the final of the five correctly answered items, with the total number of incorrect items since the basal subtracted. The PIAT reading recognition score provides an indication of a child’s ability to silently read and pronounce words. This subtest includes 84 items that require children to read and pronounce individual letters and words out loud, which increase in difficulty as the child progresses through the subtest, beginning with simple words (e.g., “run”, “play”, “jump”) and ending with more advanced words (e.g., “credulily”, “disaccharide”, “apophthegm”). Related, the PIAT reading comprehension score provides an indication of a child’s ability to read and understand full sentences or passages. This subtest includes 66 items that ask children to read a sentence silently to themselves. After they have finished reading, they are asked to point to one of four pictures that best describes what they have read. The procedures for test administration and scoring decisions for the PIAT reading comprehension and recognition subtests are identical to those described for the PIAT math scores. Thus, the PIAT reading scores are the difference between the item number of the final of the five correctly answered items and the total number of incorrect items since the basal. However, for children with a reading recognition score less than 19, the reading comprehension subset was not administered. In such cases, the reading recognition score is equal to the reading comprehension score. Further details involving norming and slight adjustments in scoring procedures over time can be found on the NLSY website. As mentioned previously, we used the raw scores (as opposed to the normed scores, used in the previously mentioned NLSYC studies) in order to investigate growth in scores across age. 

### 2.2. Statistical Models

We estimated growth curves, using multilevel modeling, for the families in the NLSY using the child PIAT scores. A family growth curve thus consisted of repeated measurements for all children in the family. Each NLSYC child had up to five repeated measurements for PIAT math, reading recognition, and reading comprehension. Multilevel models with repeated measurements at the first level, children at the second level, and mothers at the third level were estimated. We estimated models separately for the three PIAT measures, instead of adding them together as total scores, in order to detect differential family Flynn effects (as was found in, e.g., Rodgers and Wänström 2007, with regard to individual Flynn effects) for the different measures. Because, after inspection, the developmental curves showed nonlinear, quadratic growth, a quadratic age component was included in model 1:

$$\begin{array}{l} {PiatScore}_{tij}=\alpha_{ij}+\beta_{1ij}ChildAge+\beta_{2ij}{ChildAge}^{2}+\epsilon_{tij},\\ \alpha_{ij}=\alpha_{j}+v_{\alpha ij},\\ \beta_{1ij}=\beta_{1j}+v_{\beta 1ij},\\ \beta_{2ij}=\beta_{2j}+v_{\beta 2ij},\\ \alpha_{j}=\alpha +u_{\alpha j},\\ \beta_{1j}=\beta_{1}+u_{\beta 1j},\\ \beta_{2j}=\beta_{2}+u_{\beta 2j}, \end{array}$$

where ${PiatScore}_{tij}$ is the PIAT math, reading recognition, or reading comprehension score, respectively, at the *t*th age for the *i*th child of the *j*th mother, $\alpha_{ij}$ is the intercept of the growth curve for the *i*th child of the *j*th mother, $\beta_{1ij}$ is the linear slope, and $\beta_{2ij}$ is the quadratic slope of the growth curve of the *i*th child of the *j*th mother, *ChildAge* (centered around its grand mean) is the child age in months, $\epsilon_{tij}$ is a residual, $\alpha_{j}$ is the intercept for the *j*th mother, $\beta_{1j}$ is the linear slope, and $\beta_{2j}$ is the quadratic slope, for the *j*th mother, α is an overall intercept, $\beta_{1}$ and $\beta_{2}$ are overall slopes, $v_{\alpha ij},$ $v_{\beta 1ij}$ and $v_{\beta 2ij}$ are child residuals, and $u_{\alpha j},$ $u_{\beta 1j}$ and $u_{\beta 2j}$ are mother residuals. The residuals are assumed to be multivariate normally distributed within levels, and covariances between levels are assumed to be zero. Inserting the bottom equations into the top equation and collecting fixed effects at the beginning and random effects at the end yields one single equation to estimate:

$$\begin{array}{ll} {PiatScore}_{tij}= & \alpha {+\beta }_{1}ChildAge+\beta_{2}{ChildAge}^{2}{+u}_{\alpha j}+{u_{\beta 1j}ChildAge+u_{\beta 2j}{ChildAge}^{2}+v}_{\alpha ij}\\ & \;\;+v_{\beta 1ij}ChildAge{+v}_{\beta 2ij}{ChildAge}^{2}+\epsilon_{tij} \end{array}$$

We then investigated the presence of the Flynn effect in the family growth curves using the two different cohort definitions: maternal birthyear and first child birthyear. We added these cohort variables to separate models, instead of including them together in a single model. This was done because our aim was to study the effects of family cohort on family intelligence. If they were to be included together in a single model, the meaning of the cohort variables would change. The meaning of the effect of the first child birthyear, net of the effect of maternal birthyear, is for example a version of the variable maternal age at first birth. Although this is a family-level variable, it does not define membership in a cohort. 

The maternal cohort variables were added at the third level (maternal birthyear—model 2; first child birthyear—model 3):

$$\begin{array}{l} {PiatScore}_{tij}=\alpha_{ij}+\beta_{1ij}ChildAge+\beta_{2ij}{ChildAge}^{2}+\epsilon_{tij},\\ \alpha_{ij}=\alpha_{j}+v_{\alpha ij},\\ \beta_{1ij}=\beta_{1j}+v_{\beta ij},\\ \beta_{2ij}=\beta_{2j}+v_{\beta 2ij},\\ \alpha_{j}=\alpha +\gamma_{1}MaternalCohort+u_{\alpha j},\\ \beta_{1j}=\beta_{1}+\gamma_{2}MaternalCohort+u_{\beta 1j},\\ \beta_{2j}=\beta_{2}+\gamma_{3}MaternalCohort+u_{\beta 2j} \end{array}$$

A significant estimate of $\gamma_{1}$ would indicate that the family intercepts differ depending on the maternal cohort, a significant estimate of $\gamma_{2}$ would indicate that the linear part of the family slopes differs depending on the maternal cohort, and a significant estimate of $\gamma_{3}$ would indicate that the quadratic part of the family slopes differs depending on the maternal cohort. Inserting the bottom equations into the top equation to get a single equation yields:

$$\begin{array}{ll} {PiatScore}_{tij}=\alpha & +\beta_{1}ChildAge+\beta_{2}{ChildAge}^{2}+\gamma_{1}MaternalCohort+\gamma_{2}MaternalCohort\cdot ChildAge\\ & +\gamma_{3}MaternalCohort\cdot {ChildAge}^{2}+u_{\alpha j}+u_{\beta 1j}ChildAge+u_{\beta 2j}{ChildAge}^{2}+v_{\alpha ij}+v_{\beta 1ij}ChildAge\\ & +{v_{\beta 2ij}ChildAge}^{2}+\epsilon_{tij} \end{array}$$

As noted in the single equation above, two interaction terms are created from the model, and $\gamma_{1}$ can therefore also be seen as an estimate of the main effect of maternal cohort, whereas $\gamma_{2}$ and $\gamma_{3}$ can be seen as interaction effects between maternal cohort and age and age squared (note, however, that our use of the word “effect” should not be interpreted to indicate a causal relationship). 

As mentioned previously, two definitions of family cohort were used: maternal birthyear (the year the mother was born, centered around its grand mean: 1960.53) and first child birthyear (the year at which the mother had her first child, centered around its grand mean: 1983.02). A positive main effect of maternal birthyear, in the equation above, would indicate a family Flynn effect in levels, i.e., that the average family scores, at the average age of all children, and as estimated from the child PIAT scores, were higher for later born mothers. A positive interaction effect between maternal birthyear and age, on the other hand, would indicate the presence of a family Flynn effect in the slopes, i.e., that the scores of mothers born later are increasing at a higher rate, i.e., that children of mothers born later have steeper PIAT slopes over time. An interaction between maternal birthyear and age squared would indicate that the non-linear part of the developmental trajectory differs between families with mothers of different maternal birthyear cohorts. Similarly, a positive main effect of first child birthyear would indicate that children of mothers who gave birth to their first child later in time had higher estimated average scores (family Flynn effect in levels), and a positive interaction effect between first child birthyear and age (or age squared) would indicate that children of mothers who had the first child later in time were increasing at a higher rate (family Flynn effect in the slopes). Our analyses were conducted in SAS (SAS Institute Inc. 2013) version 9.4 using the procedure MIXED.

In past NLSY Flynn effect research, it has been important to adjust for an inherent selection bias in the NLSYC data. The bias is caused by older mothers with (on average) higher IQ, education, and income scores having (on average) later childbearing. Thus, children in later birth cohorts might have higher intelligence scores because of the Flynn effect, or because of this selection causing them to have higher-IQ mothers. Adjusting maternal IQ out of children’s IQ scores leaves the Flynn effect as the primary cause of any observed cohort changes in IQ. Rodgers and Wänström (2007) presented two sets of results: those for PIAT scores and those for PIAT scores adjusted for maternal cognitive ability scores on the Armed Forces Qualifying Test (AFQT), collected in the NLSY79 in 1980 when respondents were 15–22 years of age. The size of the Flynn effect reduced somewhat (but typically stayed significant) for PIAT math, and reduced to closer to zero in the PIAT reading recognition and PIAT reading comprehension scores. In the current study, this adjustment for maternal cognitive performance is not as logical for at least two reasons. First, when using maternal birthyear as the cohort measure, there is no selection bias of this type by definition (because mothers have obviously not yet given birth when they are born). 

Secondly, we are investigating possible effects of family cohort on family intelligence, as measured by child intelligence (PIAT scores). If we adjust for maternal intelligence, we are investigating the effects of family cohort on the part of child intelligence from which maternal intelligence is excluded. Although we do not find this model to be as interpretable as the models presented above, it is arguably of interest, especially when we use first child birthyear as the cohort measure. Thus, to portray these results for interested readers, we have added maternal IQ (AFQT) to Models 2 and 3 and present these results in detail in the Supplementary Materials, and more briefly here in the results section. 

## 3. Results

### 3.1. PIAT Math

The parameter estimates of fixed effects, variances of random effects, and standard errors (S.E.) from estimating Models 1, 2 and 3 for PIAT math are presented in Table 1. The estimates from Model 1 show that the estimated PIAT math score at the average age (9.75 years old) is 42.362. The estimated coefficient for the linear age slope is 0.436 and the estimated coefficient for the quadratic age slope is negative, at −0.003. The family scores are thus found to be increasing across child ages; however, the increase starts to level off for older child ages. The average linear increase is 5.232 per year (0.436 ×12), which is not a surprising number, given that children are expected to grow in their mathematical ability as they get older. There is considerable variation in the intercepts of the growth curves between families ($\sigma_{u_{\alpha j}}^{2}=32.676$), but also between the children within families ($\sigma_{v_{\alpha ij}}^{2}=24.731$). Approximately 35% of the variance in PIAT math scores is thus between-family variance (32.676/(32.676 + 24.731 + 34.682)), and approximately 27% is between children within families (24.731/(32.676 + 24.731 + 34.682)), at the average ages of the children. 

The estimates from Model 2 in Table 1 indicate a significant positive main effect of maternal birthyear. The average family score (at child age 9.75) for mothers born one year later was, on average, 0.158 higher, compared to other mothers. The usual Flynn effect is an increase of about 3 IQ points per decade (Flynn 2009). Although it is difficult to compare our estimate to the usual increase, because the PIAT tests are on a raw metric, we note that the standard deviation for PIAT scores in the NLSYC sample is approximately 10 per age in years (with lower values for younger ages, and higher values for older ages). The usual increase of 3 IQ points per decade corresponds to a 20% standard deviation (SD) increase per decade (3/15), or a 2% SD increase per year. Two percent of an SD per year corresponds to 0.2 PIAT points per year. The value 0.158 is thus a little lower than the usual individual-level Flynn effect of 3 IQ points per decade. 

**Figure 1:**
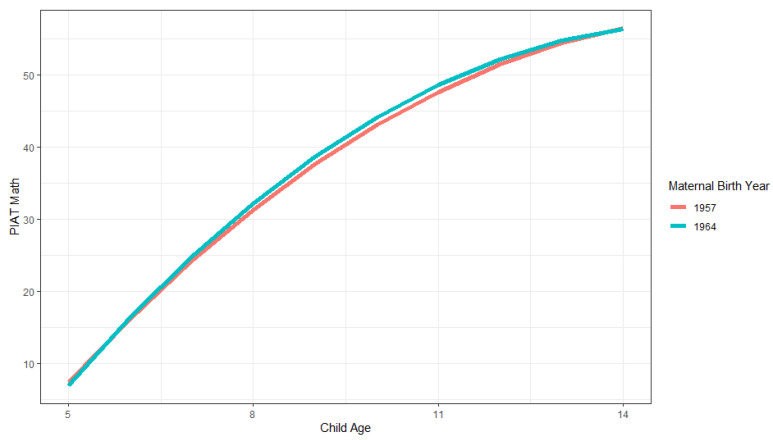
Illustration of family growth trajectories in PIAT math scores for earlier (1957) and later (1964) maternal birthyear cohorts across child ages 5 to 14.

Later-born mothers did not show a significantly steeper linear part of the slope; however, the significant quadratic component of the slope indicates that their children’s scores leveled off more with increasing ages. Figure 1 illustrates the family Flynn effects with regard to maternal birthyear. The blue line shows the model-estimated growth trajectories across child ages 5 to 14 for families of mothers born in 1964 (the latest cohort), and the red line shows the corresponding trajectory for families of mothers born in 1957 (the earliest cohort). As shown, the Flynn effect is only apparent for ages around 7–13. The model estimated difference between the cohorts is, e.g., 1.066 points for 9-year-olds, and 1.100 for 10-year-olds (which is somewhat lower than the 1.4 point difference, which would correspond to the usual Flynn effect of 3 IQ points difference per decade for these cohorts that are seven years apart).

**Figure 2:**
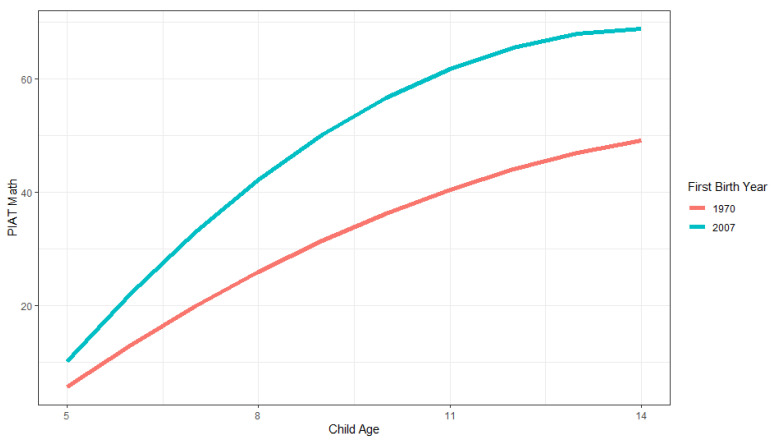
Illustration of family growth trajectories in PIAT math scores for earlier (1970) and later (2007) first child birthyear cohorts across child ages 5 to 14.

As shown from the estimates from Model 3, in Table 1, the main effect of first child birthyear is positive and significant. The average score for families who had their first child one year later is, on average, 0.543 points higher than other families, and the yearly linear increase is estimated to be 0.036 (0.003 × 12) points higher, and is estimated to level off for older ages. First child birthyear explains approximately 23.5% of the variance between families ((32.676–24.989)/32.676). Figure 2 illustrates the family Flynn effects. The blue line shows the model-estimated family trajectory for the first child birth cohort of 2007 (the latest cohort) and the red line shows the corresponding line for the 1970 cohort (the earliest cohort). As shown, child scores in the later family cohort are higher across all ages, increase more across higher ages, and then start to level off. The model-estimated differences between cohorts are about 18.701 for 9-year-olds, and 20.397 for 10-year-olds. These differences are much higher than the 7.4 point difference that would correspond to the usual Flynn effect of 3 IQ points difference per cohort decade for these cohorts that are 37 years apart.

### 3.2. PIAT Reading Recognition

The parameter estimates of fixed effects, variances of random effects, and standard errors from estimating Models 1, 2 and 3 for PIAT reading recognition are presented in Table 2. The estimated PIAT reading recognition score at the average age (9.75 years old) is 45.261, and the estimated linear increase per year is 5.652 (0.471 × 12), with a negative coefficient for the quadratic component (model 1). There is also a considerable variation in the intercepts of the growth curves, with approximately 37% of the variance in PIAT reading recognition scores between families (45.205/(45.205 + 43.789 + 32.267)), and approximately 36% between children within families (43.789/(45.205 + 43.789 + 32.267)), at the average ages of the children. 

**Figure 3:**
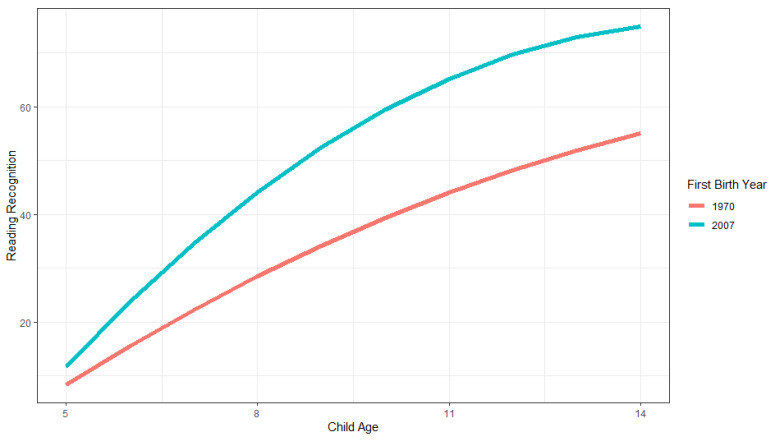
Illustration of family growth trajectories in PIAT reading recognition scores for earlier (1970) and later (2007) first child birthyear cohorts across child ages 5 to 14.

There is no significant main effect or interaction effect of maternal birthyear (Model 2). The main effect of first child birthyear and the interaction effect with age are, however, positive and significant, and the interaction with age squared is negative and significant (Model 3). The average score, at the average child age, for families who had their first child one year later is, on average, 0.529 points higher than other families. First child birthyear explains approximately 17.0% of the variance between families ((45.205–37.521)/45.205). Figure 3 illustrates these family Flynn effects. As shown, child scores in the later family cohort are higher across all ages, and increase across higher ages and then start to level off. The model-estimated differences between cohorts are about 17.980 for 9-year-olds and 19.959 for 10-year-olds, which are much higher than the 7.4 point difference that would correspond to the usual Flynn effect of 3 IQ points difference per cohort decade.

### 3.3. PIAT Reading Comprehension

The parameter estimates of fixed effects, variances of random effects, and standard errors from estimating Models 1, 2 and 3 for PIAT reading comprehension are presented in Table 3. The estimated PIAT reading comprehension score at the average age (9.75 years old) is 41.213, and the estimated linear increase per year is 4.644 (0.387 × 12) while the quadratic part of the slope is negative. There is also a considerable variation in the intercepts of the growth curves; approximately 34% of the variance in PIAT reading comprehension scores is between-family (31.751/(31.751 + 24.569 + 38.142)), and approximately 26% is between children within families (24.569/(31.751 + 24.569 + 38.142)), at the average ages of the children. 

**Figure 4:**
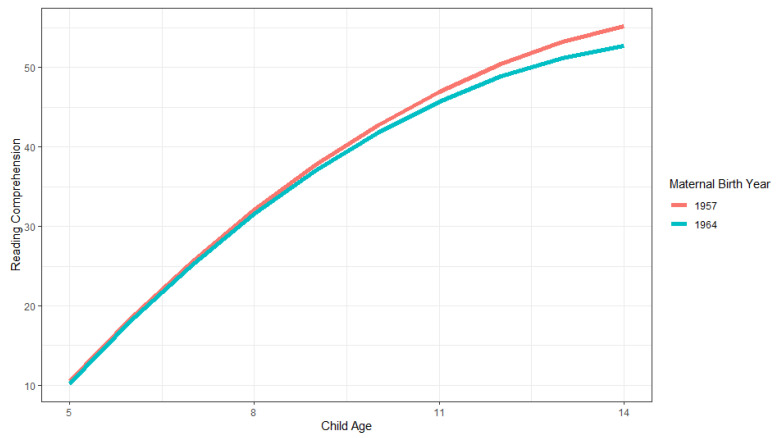
Illustration of family growth trajectories in PIAT reading comprehension scores for earlier (1957) and later (1964) maternal birthyear cohorts across child ages 5 to 14.

There is a significant negative main effect and there are significant negative interaction effects of maternal birthyear, which contradict the usual positive family Flynn effect (Model 2). These effects are illustrated in Figure 4. As shown, the scores start off at similar levels for younger ages, and then the scores of children in families in earlier maternal birthyear cohorts increase across older ages. The differences between cohorts are 0.687 for 9-year-olds and 0.926 for 10-year-olds, which increase to 2.483 for 14-year-olds. For older ages this negative Flynn effect is thus of a greater magnitude than the 1.4 difference (as expected for cohorts seven years apart). The observed standard deviations of PIAT scores for older ages were, however, larger than 10, so this latter estimate may be an overestimation.

**Figure 5:**
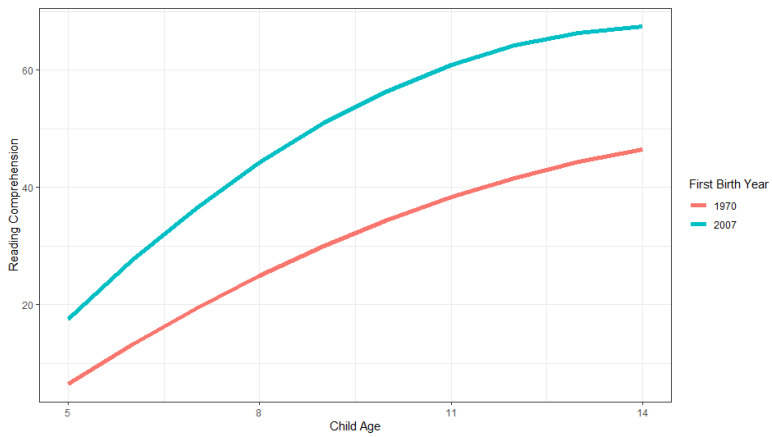
Illustration of family growth trajectories in PIAT reading comprehension scores for earlier (1970) and later (2007) first child birthyear cohorts across child ages 5 to 14.

The main effect of first child birthyear is positive and significant, as is the interaction between first child birthyear and age, whereas the interaction with age squared is negative and significant (Model 3). The average score, at the average age, for families who had their first child one year later is, on average, 0.388 points higher than other families. First child birthyear explains approximately 12.9% of the variance between families ((31.751–27.644)/31.751). Figure 5 illustrates these family Flynn effects. As shown, the child scores in the later family cohort are higher across all ages, and increase across higher ages and then start to level off. The model-estimated differences between cohorts are about 20.863 for 9-year-olds, and 21.964 for 10-year-olds, which are much higher than the 7.4 point difference that would correspond to the usual Flynn effect of 3 IQ points difference per cohort decade.

### 3.4. Adjusting for Maternal IQ

When we added maternal IQ to Models 2 and 3, with PIAT math, reading recognition, and reading comprehension as response variables, the main effects of maternal birthyear and first child birthyear typically decreased, but stayed significant, in the models in which they were previously significant. None of the interaction effects changed much. The main effect of maternal birthyear decreased from 0.158 to 0.102 for PIAT math, and from −0.124 to −0.170 for PIAT reading comprehension. The main effect of first child birthyear decreased from 0.543 to 0.307 for PIAT math, from 0.529 to 0.227 for PIAT reading recognition, and from 0.388 to 0.153 for PIAT reading comprehension. The effect of maternal IQ was positive and significant in all models, as expected, and decreased the between-family variance (also as expected). A complete table with results adjusting for maternal IQ can be found in the Supplementary Materials.

### 3.5. Summary of the Results

We found Flynn effects for both of our cohort definitions. With regard to the first cohort definition, maternal birthyear, we found family Flynn effects in PIAT math scores of a slightly smaller magnitude than the usual individual Flynn effect magnitude found in previous studies. Children in families in which the mother was born later started off (at age 5) at about the same level as children in families in which the mother was born earlier, then increased in terms of math scores at a higher rate, before leveling off to about the same levels at ages 14. We also found family cohort effects of maternal birthyear in PIAT reading comprehension; however, these were in the opposite direction (sometimes referred to as reverse Flynn effects). Children in families in which the mother was born later started off (at age 5) at about the same level as children in families in which the mother was born earlier; however, these then had a slower development, and differed by more than the usual individual Flynn effect magnitude by the age of 14. 

With regard to the second cohort definition, first child birthyear, we found family Flynn effects of higher magnitudes compared to the Flynn effects for maternal birthyear. Children in families that had their first child later had higher PIAT math, reading recognition, and reading comprehension scores than children in families that had their first child earlier overall, but they also increased in terms of scores at a higher rate. The differences in increases started leveling off somewhat for older ages. The patterns were similar for math, reading recognition, and reading comprehension scores, and these family cohort effects were much larger than the usual individual Flynn effect magnitude. 

## 4. Discussion

The aim of our study was to investigate the presence of family-level Flynn effects, in levels and in growth. We used child PIAT raw scores and multilevel growth models to obtain estimates of family-level intelligence scores in the NLSY79 and NLSYC data. Previous studies found child Flynn effect patterns, particularly in the NLSYC PIAT math measures (Rodgers and Wänström 2007; Ang et al. 2010). Following this, O’Keefe and Rodgers (2017) identified between-family variance as the primary source of the child Flynn effect in the NLSYC PIAT math scores. Our design differs from those because we evaluated the Flynn effect longitudinally using raw scores, and studied the effects on both family levels and family slopes, using family cohort measures (rather than child cohort measures). It is not as straightforward to define family cohorts as individual cohorts. Our first definition, the mother’s birthyear, assumes that the family entity is influenced by the mother’s own cohort. Our other definition, the birthyear of the first child, assumes that the family entity starts when the first child is born. Our choices of family cohort measures fit our data well, because the NLSYC data contain information that help us to easily construct these measures. Other definitions are possible and may fit other data sources better. 

The rate of development may be different for different cohorts, but it may also be different for different age intervals, and/or time intervals, making longitudinal assessments of the Flynn effect important. Our use of raw scores enabled us to estimate both the levels and slopes of growth curves. We found significant variance both within individuals (individual child scores increase as the children get older, due to an ageing/growth effect), between individuals within families (differences in scores of children in the same family), and between families (differences in scores of children of different families). Our design enabled us to find both Flynn effects in the average scores of families of different cohorts (levels) and differences in family growth (slopes). Mothers born later, on average, had children who increased more in terms of their math scores between the ages of 7 and 13, with children of mothers born earlier then catching up at later ages. Children of mothers born later, however, increased in terms of reading comprehension scores at a slightly lower rate compared to children of mothers born earlier. Mothers who started their families later had steeper increases in all three subtests, although the difference in increases leveled off at older ages. 

There is no consensus in the research community as to the causes of the Flynn effect. Most researchers consider multiple causes (see, for example, Pietschnig and Voracek 2015, among others). Flynn effects on growth, as found in our study, have implications for the search for explanations of the Flynn effect. Explanations should explain differences in levels, but also in growth. For example, parents with higher education levels may be able to continue to help their children with schoolwork as they grow older. Improved child education, or equal opportunity programs, could be other explanatory factors. Moreover, family Flynn effects may have different interpretations compared to individual Flynn effects. The presence of individual Flynn effects suggests that the individual is somehow affected by the time in which he/she is born and grows up, e.g., that time-linked improvements in nutrition, education, etc., are beneficial to individual cognition. Family Flynn effects, on the other hand, suggest that the overall family changes in a time-related context (because of the family providing better nutrition to the children, Lynn 2009; or the parents having better education, Cuartas 2022, etc.), and that this context is important to the cognition of the family members. 

Our results differ between family cohort definitions. Explanations of our maternal birthyear effect should be sought in factors that differ between mothers of different birth cohorts, e.g., education and income, nutrition, or maternal Flynn effects themselves. In other words, the usual theories used to explain individual-level Flynn effect findings from previous studies are relevant. Overall, our observed family Flynn effects were consistently larger for the first child birthyear cohort definition than for the maternal birthyear definition, suggesting that the family Flynn effect leverages off of both the mother and the children in the family. The timing of parents’ entry into parenthood appears to be more important for these family outcomes than parental cohort. Adding first child birthyear to the growth curve models also explained substantive parts of the between-family variance in PIAT scores. We found that mothers who had their first child later had children who increased faster in terms of their scores, although the difference between cohorts started to level off with older ages. The usual Flynn effect of 3-IQ points increase per decade corresponds to a 20% SD increase. Our observed first child birthyear effect was around a 50% SD increase per decade for 9-year-olds, and this became larger as children grew older (however, it leveled off around age 14). Explanations of this family Flynn effect should be sought in family-level factors/events that differ between time points after the first child is born, e.g., better education or parental caregiving systems for the children in families who start later, higher incomes, higher standards of living, etc. Ang et al. (2010) examined sub-group Flynn effects for the NLSYC PIAT math test, and found that children in higher-income families, and children with higher-educated mothers, had stronger Flynn effects. Shakeel and Peterson (2022) found larger Flynn effects for higher SES groups among older students; however, they found smaller effects for higher SES groups among younger students (which they note may have been due to equal opportunity programs). The relationship between SES and cognitive ability is well-known (e.g., McCulloch and Joshi 2001; Nash 2001; Neiss et al. 2002; Paterson 2021; Rodgers et al. 2008), and an obvious explanation of our results may therefore be that higher-IQ mothers (and higher SES families) tend to start their families later. Our family Flynn effects decreased somewhat, but still persisted after controlling for maternal IQ, suggesting that this is not the sole explanation, however. 

We feel that one of the under-appreciated explanations for the Flynn effect is quality of parenting, and this interpretation may help explain some of our findings regarding the increasing differences for children of different family cohorts, which start to level off at older ages. It seems virtually axiomatic that the effect of parenting matters the most for young children, and then reduces as children get older. O’Keefe and Rodgers (2022) evaluated secular changes in the quality of the home environment in the NLSY-Children data, and found results consistent with this interpretation. For children from infancy through age 9, the quality of the home environment had been improving between 1985 and 2010. However, as these children aged into adolescence, this secular change weakened and disappeared. We stress, however, that our purpose in the current study was foremost to test for family Flynn effects in our data, and not to search for possible causes of the effects. We call for future research to search for nuanced family-level explanations/causes (as detection necessarily preceeds the search for explanations). Future research may estimate family growth models and add factors at different levels (factors that change over time, factors that differ between individuals, and factors that differ between families). Several of the factors mentioned in the previous paragraph are available in the NLSY79 and the NLSYC data.

As noted previously, our observed family Flynn effects were smaller in magnitude for maternal birthyear than for the first child birthyear cohort definition. They were also inconsistent between subtests for the maternal birthyear definition. We found family Flynn effects in PIAT math of about a 15.2% SD increase for children around 9 years old. Although smaller than our other cohort definition, this magnitude is similar to those in Trahan et al.’s (2014) meta-analysis, with an overall Flynn effect of 2.31 (=15.4% SD) when including all available Flynn effect studies, and an effect of 2.93 (=19.5% SD) when including recent studies using Wechsler or Binet tests. Our results show that mothers born later, on average, had children who showed greater increases in their PIAT math scores between the ages of 7 and 13, with children of mothers born earlier then catching up at later ages, suggesting that this PIAT math family Flynn effect in these data primarily occurred in young and middle childhood. This goes along with the results of Shakeel and Peterson (2022), who found larger child math Flynn effects at younger ages (around age 9) compared to early adolescence (around 13–15) and older ages (around 17). We found no Flynn effect for PIAT reading recognition, and a negative (reverse) Flynn effect for PIAT reading comprehension. The magnitude of this negative Flynn effect was about 9.8% SD for 9-year-olds, increasing as the children grew older. Differential effects for math and reading scores, as well as negative Flynn effects, have also been found previously. Shakeel and Peterson (2022) found Flynn effects (differing by the agency administering the test) varying from a 10% SD decrease to a 27% SD increase for math tests, and from a 2% SD decrease to a 12% SD increase for reading tests. Rodgers and Wänström (2007) found positive child Flynn effects for PIAT math scores and reading comprehension scores; however, the positive child Flynn effects for raw reading comprehension scores decreased to a mean value of 0 for child ages 0–13 when maternal IQ was controlled. Individual Flynn effects have usually been larger for fluid than for crystallized intelligence (Pietschnig and Voracek 2015), and stronger correlations have been found between fluid tests and math tests than between fluid tests and reading tests (Peng et al. 2019), which may account for some of the differences between our observed effects. Some items in the PIAT math subtest are more related to problem-solving than the items in the reading recognition and reading comprehension subtests. Ang (2008) performed an item analysis of the PIAT math test and found that items that were more related to problem solving contained more of a Flynn effect than others. These were items such as “Two birds were on a fence. Two more landed on the fence. How many birds were now on the fence?” (note that this is not a real PIAT math item, but rather simply an example). In contrast, almost equivalent items such as “what is 2+2?” did not contain as much of a Flynn effect. More studies are, however, needed to examine whether our negative Flynn effect result is specific for the test (PIAT reading comprehension), for the family cohort definition (maternal birthyear as opposed to first child birthyear), or for the dataset (NLSYC).

We have not investigated the presence of Flynn effects at the individual child level in our study. We know that they exist (Ang et al. 2010; O’Keefe and Rodgers 2017; Rodgers and Wänström 2007); however, adding a child cohort variable into a model with a family cohort variable changes the interpretation of the family cohort variable. For example, the effect of first child birthyear, net of the effect of maternal birthyear, is a version of the variable maternal age. These types of analyses can, however, be found in O’Keefe and Rodgers (2017), who separated cohort variables into within-child, between-child, and between-family measures, and related them to PIAT math scores. We also were not able to examine Flynn effects on maternal cognition in the NLSY data, because cognition was only measured at one time point for the NLSY79 sample, and the mothers were of varying ages (see Rodgers and Wänström 2007). Future studies, using other data sources, may be able to disentangle parental Flynn effects and family Flynn effects, although such complex longitudinal datasets may be difficult to identify. 

In summary, several contributions are made to the Flynn effect literature within the current study. We have focused on Flynn effects in the family (motivated by previous NLSY studies, in particular O’Keefe and Rodgers 2017). In addition, we examined Flynn effects on raw score levels and on growth. Future research could focus on replicating our results in other datasets, expanding to different age intervals, disentangling individual and family Flynn effects, and examining possible explanatory factors at different levels (within-person, between persons, and between families).

## Figures and Tables

**Table 1:** Estimates, variances and standard errors (S.E.) from three-level models with PIAT math as response variable.

	Model 1		Model 2		Model 3	
	Estimate	S.E.	Estimate	S.E.	Estimate	S.E.
Fixed Effects						
Intercept	42.362 ***	0.119	42.393 ***	0.119	42.268 ***	0.110
ChildAge	0.436 ***	0.001	0.435 ***	0.001	0.433 ***	0.001
ChildAge^2^	−0.003 ***	0.00003	−0.003 ***	0.00003	−0.003 ***	0.00003
Maternal birthyear			0.158 **	0.053		
First child birthyear					0.543 ***	0.019
ChildAge × Maternal birthyear			−0.00006	0.0006		
ChildAge^2^ × Maternal birthyear			−0.00007 ***	0.00001		
ChildAge × First child birthyear					0.003 ***	0.0002
ChildAge^2^ × First child birthyear					−0.00007 ***	0.000005
Random effects	Variance	S.E.	Variance	S.E.	Variance	S.E.
$u_{\alpha j}$	32.676 ***	1.145	32.653 ***	1.144	24.989 ***	0.978
$u_{\beta 1j}$	0.002 ***	0.0001	0.002 ***	0.0001	0.002 ***	0.0002
$v_{\alpha ij}$	24.731 ***	0.689	24.739 ***	0.689	24.886 ***	0.689
$v_{\beta 1ij}$	0.002 ***	0.0002	0.002 ***	0.0002	0.002 ***	0.0002
$\epsilon_{tij}$	34.682 ***	0.363	34.653 ***	0.362	34.444 ***	0.360
-2ResLogLikelihood	239,280.1		239,280.8		238,350.7	

** *p* > .01, *** *p* < .001. Note. Models with random effects of quadratic slopes failed to converge, and those random effects were therefore omitted.

**Table 2:** Estimates, variances and standard errors (S.E.) from three-level models with PIAT reading recognition as response variable.

	Model 1		Model 2		Model 3	
	Estimate	S.E.	Estimate	S.E.	Estimate	S.E.
Fixed Effects						
Intercept	45.261 ***	0.140	45.268 ***	0.140	45.169 ***	0.133
ChildAge	0.471 ***	0.002	0.471 ***	0.002	0.468 ***	0.002
ChildAge^2^	−0.003 ***	0.00003	−0.003 ***	0.00003	−0.003 ***	0.00003
Maternal birthyear			0.029	0.063		
First child birthyear					0.529 ***	0.022
ChildAge × Maternal birthyear			−0.00069	0.0008		
ChildAge^2^ × Maternal birthyear			−0.00002	0.00001		
ChildAge × First child birthyear					0.004 ***	0.0003
ChildAge^2^ × First child birthyear					−0.00007 ***	0.000005
Random effects	Variance	S.E.	Variance	S.E.	Variance	S.E.
$u_{\alpha j}$	45.205 ***	1.642	45.231 ***	1.643	37.521 ***	1.478
$u_{\beta 1j}$	0.005 ***	0.0003	0.005 ***	0.0003	0.005 ***	0.0003
$v_{\alpha ij}$	43.789 ***	1.053	43.790 ***	1.053	44.029 ***	1.056
$v_{\beta 1ij}$	0.006 ***	0.0003	0.006 ***	0.0003	0.006 ***	0.0003
$\epsilon_{tij}$	32.267 ***	0.345	33.265 ***	0.345	32.064 ***	0.343
-2ResLogLikelihood	243,485.8		243,518.2		242,852.5	

*** *p* < .001. Note. Models with random effects of quadratic slopes failed to converge, and those random effects were therefore omitted.

**Table 3:** Estimates, variances and standard errors (S.E.) from three-level models with PIAT reading comprehension as response variable.

	Model 1		Model 2		Model 3	
	Estimate	S.E.	Estimate	S.E.	Estimate	S.E.
Fixed Effects						
Intercept	41.213 ***	0.119	41.219 ***	0.119	41.1578 ***	0.115
ChildAge	0.387 ***	0.002	0.387 ***	0.002	0.385 ***	0.002
ChildAge^2^	−0.003 ***	0.00003	−0.003 ***	0.00003	−0.003 ***	0.00004
Maternal birthyear			−0.124 *	0.053		
First child birthyear					0.388 ***	0.020
ChildAge × Maternal birthyear			−0.003 ***	0.0007		
ChildAge^2^ × Maternal birthyear			−0.00003 *	0.00002		
ChildAge × First child birthyear					0.002 ***	0.0003
ChildAge^2^ × First child birthyear					−0.00005 ***	0.000006
Random effects	Variance	S.E.	Variance	S.E.	Variance	S.E.
$u_{\alpha j}$	31.751 ***	1.141	31.672 ***	1.1397	27.644 ***	1.059
$u_{\beta 1j}$	0.003 ***	0.0002	0.003 ***	0.0002	0.003 ***	0.0002
$v_{\alpha ij}$	24.569 ***	0.717	24.539 ***	0.716	24.820 ***	0.722
$v_{\beta 1ij}$	0.002 ***	0.0002	0.002 ***	0.0002	0.002 ***	0.0002
$\epsilon_{tij}$	38.142 ***	0.409	38.149 ***	0.409	38.066 ***	0.408
-2ResLogLikelihood	236,493.8		236,498.1		236,090.9	

* *p* < .05, *** *p* < .001. Note. Models with random effects of quadratic slopes failed to converge, and those random effects were therefore omitted.

## Data Availability

We used a public data source (available here: https://www.nlsinfo.org/ accessed on 1 October 2022), and our analyses were conducted in SAS version 9.4 using available procedures MIXED.

## Supplementary Materials

The following supporting information can be downloaded at: https://www.mdpi.com/article/10.3390/jintelligence11030050/s1, Table S1: Estimates and variances from three-level models controlling for maternal IQ.
