# Supplementary material for: It Runs in the Family: Testing for Longitudinal Family Flynn Effects
Source: J Intell. 2023 Mar 6;11(3):50. doi: 10.3390/jintelligence11030050 (PMC10057072; doi:10.3390/jintelligence11030050)
Supplement: Supplementary file 1 [file jintelligence-11-00050-s001.zip › jintelligence-2038283-supplementary.pdf]

Table S1 shows parameter estimates of fixed effects and variances of random effects from estimating models 2 and 3 when maternal IQ (mothers' AFQT scores) have been added. As shown in the first column, the main effect of maternal birthyear on PIAT math is 0.102 (previously 0.158) and significant. The interaction effect between maternal birthyear and child age squared is negative and significant. This suggests that the pattern found previously (when maternal IQ was not part of the model) holds when maternal IQ is controlled for, however with a smaller difference between maternal birthyear cohorts, which is not surprising since maternal IQ and child IQ are positively correlated. The variance between family levels decreases by more than 50%, from 32.653 to 15.605, when maternal IQ is added. Similar patterns are shown in the other models with the other family cohort definition (first child birthyear) and with the other response variables (reading recognition and reading comprehension). The main effects decrease and stay significant (from -0.124 to -0.170 for maternal birthyear and reading comprehension; from 0.543 to 0.307 for first child birthyear and math; from 0.529 to 0.227 for first child birthyear and reading recognition; and from 0.388 to 0.153 for first child birthyear and reading comprehension) while the interaction effects are about the same as before. The effect of maternal IQ is positive and significant in all models, as expected, and the variance in family level scores decrease substantially when maternal IQ is added.

Table S1. Estimates and variances from three-level models controlling for maternal IQ

	PIAT math (n=33401)		PIAT RR (n=33269)		PIAT RC (n=32592)	
	Model 2	Model 3	Model 2	Model 3	Model 2	Model 3
Fixed Effects	Estimate	Estimate	Estimate	Estimate	Estimate	Estimate
Intercept	42.352***	42.324***	42.204***	45.190***	41.154***	41.169***
ChildAge	0.436***	0.434***	0.472***	0.469***	0.388***	0.386***
ChildAge^2	-0.003***	-0.003***	-0.003***	-0.003***	-0.003***	-0.003***
Maternal birthyear	0.102*		-0.021		-0.170***	
First child birthyear		0.307***		0.277***		0.153***
ChildAge x Maternal birthyear	-0.00007		-0.001		-0.003***	
ChildAge^2 x Maternal birthyear	-0.00008***		-0.00002		-0.00003*	
ChildAge x First child birthyear		0.003***		0.004***		0.002***
ChildAge^2 x First child birthyear		-0.00007***		-0.00006***		-0.00005***
Maternal IQ	0.0001***	0.0001***	0.0002***	0.0001***	0.0001***	0.0001***
Random effects	Variance	Variance	Variance	Variance	Variance	Variance
$u_{\alpha j}$	15.605***	14.157***	26.375***	25.050***	16.245***	16.106***
$u_{\beta 1j}$	0.002***	0.002***	0.005***	0.005***	0.003***	.003***
$v_{\alpha ij}$	24.942***	24.949***	43.819***	43.917***	24.662**	24.835***
$v_{\beta 1ij}$	0.002***	0.002***	0.006***	0.006***	0.002***	.002***
ϵ_{tij}	34.672***	34.501***	32.264***	32.055***	38.241***	38.162***
-2ResLogLikelihood	230167.8	229691.3	234610.2	234274.7	227700.8	227583.6

*p<.05, **p>.01 ***p<.001

Note. Models with random effects of quadratic slopes failed to converge, and those random effects were therefore omitted.
